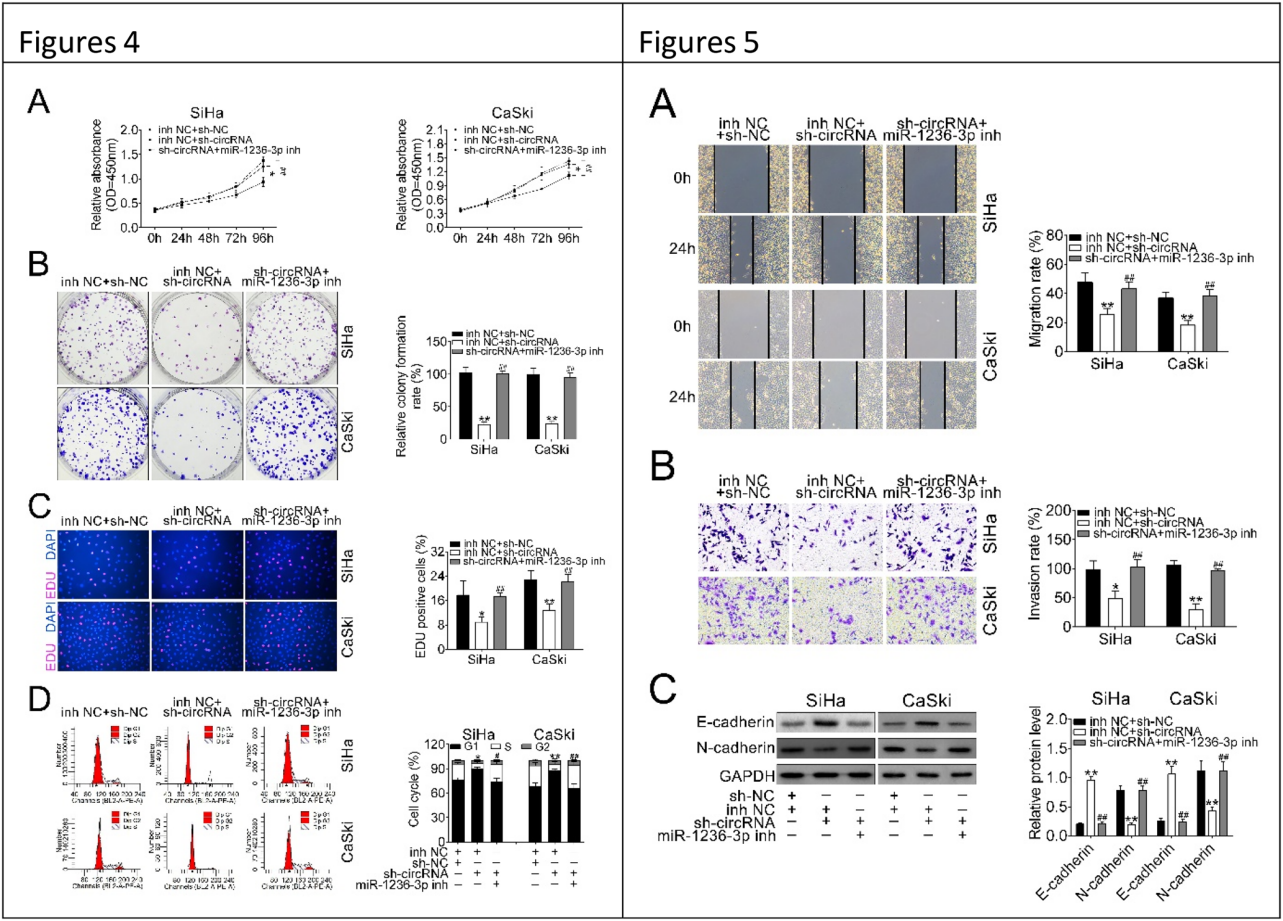# Correction to “Circular RNA circRNA_101996 Promoted Cervical Cancer Development by Regulating miR‐1236‐3p/TRIM37 Axis”

**DOI:** 10.1002/kjm2.70151

**Published:** 2025-12-05

T. F. Song, A. L. Xu, X. H. Chen, J. Y. Gao, F. Gao, and X. C. Kong, “Circular RNA circRNA_101996 Promoted Cervical Cancer Development by Regulating miR‐1236‐3p/TRIM37 Axis,” *Kaohsiung Journal of Medical Sciences* 37 (2021): 547–561, https://doi.org/10.1002/kjm2.12378.

**Table:** 

We identified an inadvertent image duplication and intra‐group mixing in Figures 4B and 5B of our published article. The corrected images are shown below. This error occurred during figure assembly and does not affect the experimental results or conclusions. We sincerely apologize for the oversight and request that the correction be made accordingly. In addition, the details of the changes were below: All six photos in Fig 4B have been replaced. All six photos in Fig 5B have been replaced. The authors confirmed that all results and conclusions of this article remain unchanged. We apologize for this error.